# Cationic Net Charge and Counter Ion Type as Antimicrobial Activity Determinant Factors of Short Lipopeptides

**DOI:** 10.3389/fmicb.2017.00123

**Published:** 2017-02-01

**Authors:** Katarzyna E. Greber, Malgorzata Dawgul, Wojciech Kamysz, Wieslaw Sawicki

**Affiliations:** ^1^Department of Physical Chemistry, Faculty of Pharmacy, Medical University of GdanskGdansk, Poland; ^2^Department of Inorganic Chemistry, Faculty of Pharmacy, Medical University of GdanskGdansk, Poland

**Keywords:** lipopeptides, antimicrobial activity, MIC, MBC, TFA counter ion, fatty acid chain length

## Abstract

To get a better insight into the antimicrobial potency of short cationic lipopeptides, 35 new entities were synthesized using solid phase peptide strategy. All newly obtained lipopeptides were designed to be positively charged from +1 to +4. This was achieved by introducing basic amino acid - lysine - into the lipopeptide structure and had a hydrophobic fatty acid chain attached. Lipopeptides were subjected to microbiological tests using reference strains of Gram-negative bacteria: *Escherichia coli, Klebsiella pneumoniae, Proteus vulgaris, Pseudomonas aeruginosa*, Gram-positive bacteria: *Staphylococcus aureus, Staphylococcus epidermidis, Bacillus subtilis, Enterococcus faecalis*, and fungi: *Candida albicans, Candida tropicalis, Aspergillus brasiliensis*. The minimum inhibitory concentration (MIC), minimum bactericidal concentration (MBC) and minimal fungicidal concentration (MFC) were established for each strain. The toxicity toward human cells was determined by hemolysis tests via minimum hemolytic concentration (MHC) determination. The effect of the trifluoroacetic acid (TFA) counter ion on the antimicrobial activity of lipopeptides was also examined by its removing and performing the antimicrobial tests using counter ion-free compounds. The study shows that lipopeptides are more potent against Gram-positive than Gram-negative strains. It was revealed that positive charge equals at least +2 is a necessary condition to observe significant antimicrobial activity, but only when it is balanced with a proper length of hydrophobic fatty acid chain. The hemolytic activity of lipopeptides strongly depends on amino acid composition of the hydrophilic portion of the molecule as well as fatty acid chain length. Compounds endowed with a greater positive charge were more toxic to human erythrocytes. This should be considered during new lipopeptide molecules design. Our studies also revealed the TFA counter ion has no significant effect on the antimicrobial behavior of cationic lipopeptides.

## Introduction

Most naturally occurring antimicrobial peptides are positively charged and their amino acid sequences form an amphipathic structure. These structural conditions seem to be mandatory for the occurrence of high levels of antimicrobial activity (Hancock and Diamond, 2000; Brown and Hancock, 2006; Hancock and Sahl, 2006). Therefore, antimicrobial peptide molecule design and the synthesis of positively charged antimicrobial peptide compounds are often achieved by introducing basic amino acids, e.g., lysine or arginine, into the sequence. In turn, the condition of amphipathicity is provided by introducing amino acids with hydrophobic side chains. It is also possible to create amphiphilic peptide-like molecule by replacing a few hydrophobic amino acid residues with one, also hydrophobic, fatty acid chain. (Makovitzki et al., 2006; Domalaon et al., 2016). A large group of potent antimicrobial lipopeptides have been generated this way. Some of them, e.g., surotomycin, dalbavancin and HB1345, are currently being developed by the pharmaceutical industry and are undergoing clinical trials (Greber and Dawgul, 2016). The emergence and spread of multidrug resistant (MDR) microorganisms causes that a development of a new antibiotics is nowadays one of the most important challenges of pharmaceutical chemistry (Ventola, 2015). Due to the issue of researching of an effective antibiotic therapies is urgent, we decided to synthesize a group of potentially antimicrobial cationic lipopeptides and study their antibacterial and antifungal potency. Our lipopeptides are divided into five groups differing in fatty acid chain length and cationic net charge. In the present study, we determined the antibacterial and antifungal potency of lipopeptides toward 11 reference strains of Gram-negatives: *Escherichia coli, Klebsiella pneumoniae, Proteus vulgaris, Pseudomonas aeruginosa*, Gram-positives: *Staphylococcus aureus, Staphylococcus epidermidis, Bacillus subtilis, Enterococcus faecalis*, and fungi: *Candida albicans, Candida tropicalis*, and *Aspergillus brasiliensis*. The minimum inhibitory concentration (MIC), minimum bactericidal concentration (MBC) and minimal fungicidal concentration (MFC) were established for each strain. The toxicity toward human cells was tested via minimum hemolytic concentration (MHC) determination. Solid phase peptide synthesis procedure, employed in this work, as well as chromatographic purification of lipopeptides requires use of trifluoroacetic acid. This means that cationic lipopeptides are obtained as trifluoroacetate salts. Due to the fact that trifluoroacetic ion is not a physiological and it is not present in the common microbial environment it may have an impact on microbial growth. We decided to study if there is a difference in antimicrobial activity of lipopeptides in the form of trifluoroacetic salts and lipopeptides in the form of bases. There is no other data published in this matter.

## Materials and methods

### Chemicals

All chemicals and solvents for peptide synthesis were from Iris Biotech (Germany). The column for lipopeptide RP-HPLC purification (Nucleodur C8e/c, 100 Å, 5 μm, 250 × 10 mm) was from Macherey-Nagel (Germany). The analytical column for peptide characterization (Chromolith Performance RP-18e, 100 × 4.6 mm) was from Merck Millipore (Germany). The gradient grade acetonitrile was from Scharlau (Spain). Deionized water (18.2 M'Ω) was obtained using a Direct-Q 3 UV-R Ultrapure Water System, Merck-Millipore, (Germany). The ion exchange device for TFA removal was a VariPure IPE (Ion Pair Extraction), Agilent (USA).

### Peptide synthesis and purification

All lipopeptides were synthesized using the Fmoc/tBu procedure on Fmoc-Rink Amide AM resin (0.59 mmol/g) (Table 1). The Fmoc protecting group of amino acid was detached by 20% piperidine in DMF. Peptide bond formation was carried out with a 3-fold excess of Fmoc-protected amino acid dissolved in a DMF/DCM mixture (1:1 v/v) in the presence of 1% of Triton X-100 using DIC and HOBt as coupling reagents. Removal of the side chain protecting group and cleavage from the resin was achieved via 95% TFA with 2.5% of TIS and 2.5% of water for 1 h (Chan and White, 2000). The cleaved lipopeptides were precipitated and lyophilized. They were then purified by semi-preparative reverse-phase high performance liquid chromatography (RP-HPLC) with a Macharey Nagel C8 e/c column and eluted with a 20–60% linear gradient of phase B (phase A—0.1% TFA in water, phase B—0.1% TFA in ACN), at a flow rate of 3 mL/min, and analyzed at 214 nm.

**Table 1:** **Physicochemical characterization of synthesized cationic lipopeptides**.

**Group number**	**Denotation**	**Fatty acid chain**	**Net charge**	**Molecular mass**		**Retention time t_R_**
				**Calculated**	**Observed**	
1	C_16_-K-NH_2_	Heksadecanoic	+1	383.4	384.4	8.17
	C_16_-KK-NH_2_		+2	511.5	512.5	7.02
	C_16_-KKK-NH_2_		+3	639.5	640.5	6.58
	C_16_-KKKK-NH_2_		+4	767.6	768.6	6.20
	C_16_-KG-NH_2_		+1	440.4	441.4	8.24
	C_16_-KGK-NH_2_		+2	568.4	569.4	7.07
	C_16_-KGKG-NH_2_		+2	625.5	626.5	7.10
2	C_14_-K-NH_2_	Tetradecanoic	+1	355.4	356.4	7.22
	C_14_-KK-NH_2_		+2	483.3	484.3	6.33
	C_14_-KKK-NH_2_		+3	611.5	612.5	5.93
	C_14_-KKKK-NH_2_		+4	739.2	740.2	5.57
	C_14_-KG-NH_2_		+1	412.4	413.4	7.00
	C_14_-KGK-NH_2_		+2	540.2	541.2	6.25
	C_14_-KGKG-NH_2_		+2	597.4	598.4	6.31
3	C_12_-K-NH_2_	Dodecanoic	+1	327.2	328.2	6.44
	C_12_-KK-NH_2_		+2	455.6	456.6	5.43
	C_12_-KKK-NH_2_		+3	583.2	584.2	4.92
	C_12_-KKKK-NH_2_		+4	711.2	712.2	4.37
	C_12_-KG-NH_2_		+1	384.4	385.4	6.52
	C_12_-KGK-NH_2_		+2	512.2	513.2	5.20
	C_12_-KGKG-NH_2_		+2	569.2	570.2	5.42
4	C_10_-K-NH_2_	Decanoic	+1	299.3	300.3	5.22
	C_10_-KK-NH_2_		+2	427.3	428.3	4.57
	C_10_-KKK-NH_2_		+3	555.5	556.5	4.33
	C_10_-KKKK-NH_2_		+4	683.3	684.3	4.00
	C_10_-KG-NH_2_		+1	356.3	357.3	5.31
	C_10_-KGK-NH_2_		+2	484.2	485.2	4.45
	C_10_-KGKG-NH_2_		+2	541.3	542.3	4.68
5	C_8_-K-NH_2_	Octanoic	+1	271.2	272.2	4.33
	C_8_-KK-NH_2_		+2	399.3	400.3	3.54
	C_8_-KKK-NH_2_		+3	527.2	528.2	3.28
	C_8_-KKKK-NH_2_		+4	655.4	656.4	3.11
	C_8_-KG-NH_2_		+1	328.3	329.3	4.24
	C_8_-KGK-NH_2_		+2	456.4	457.4	3.58
	C_8_-KGKG-NH_2_		+2	513.1	514.1	5.50

### Reversed-phase analysis of lipopeptides

The lipopeptides were analyzed by RP-HPLC on a Chromolith Performance monolithic column with a linear gradient 2–98% phase B (where phase A was 0.1% TFA in water and phase B was 0.1% TFA in ACN), at a flow rate of 2 mL/min, and analyzed at 214 nm. Fractions with a purity greater than 95% were collected and freeze-dried on an Alpha 2–4 LD plus system (Christ, Germany). Lipopeptides were further characterized by matrix assisted laser desorption ionization time-of-flight mass spectrometry (MALDI-TOF, Bruker, Germany).

### Organisms and antimicrobial assay

The minimum inhibitory concentration (MIC), minimum bactericidal concentration (MBC) and minimum fungicidal concentration (MFC) were determined according to the procedure recommended by the Clinical Laboratory Standards Institute (CLSI). The following reference strains were tested: Gram-negative *E. coli* (ATCC 25922), *K. pneumoniae* (ATCC 700603), *P. vulgaris* (PCM 2668), *P. aeruginosa* (ATCC 9027), Gram-positive *S. aureus* (ATCC 25923), *S. epidermidis* (PCM 2118), *B. subtilis* (ATCC 6633), *E. faecalis* (ATCC 29212) and fungi *C. albicans* (ATCC 10231), *C. tropicalis* (PCM 2681), and *A. brasiliensis* (ATCC 16404). All the microorganisms were from the Polish Collection of Microorganisms (Wroclaw, Poland). MIC was determined using a microbroth dilution method with Mueller-Hinton (MH) broth for bacteria and Sabouraud Dextrose broth for fungi (Becton Dickinson, Le Pont de Claix, France). An initial inoculum of 10^5^ cfu/mL was used for bacteria and 10^3^ cfu/mL for fungi. Polypropylene 96-well plates (Kartell, Noviglio, Italy) were incubated for 18 h at 37°C for bacteria and for 48 h at 25°C for the tested fungi. MIC (μg/mL) was taken as the lowest concentration of the lipopeptide, at which the observable growth was inhibited. All the MIC wells which did not reveal turbidity were cultured on solid media: a Mueller-Hinton II Agar for bacteria and a Sabouraud 2% Glucose Agar for fungi. The lowest concentrations of lipopeptides that did not show any visible growth on the plates after 24 h of incubation at 37°C and 48 h of incubation at 25°C were recorded as MBCs and MFCs, respectively. The experiments were performed in triplicate on three different days.

### Hemolysis

The hemolytic activity of the lipopeptides was determined by exposing human red blood cells (4% v/v) to tested compounds at graded concentrations. Red blood cells were obtained from a healthy donor. EDTA was used to prevent the blood clotting. The plasma was removed via centrifugation and the erythrocytes were washed three times with PBS. The red blood cells were suspended in PBS and incubated with different concentrations of lipopeptides at 37°C for 1 h and centrifuged for 5 min at 1000 × g. The supernatants were transferred to a sterile 96-well plate and hemoglobin release was measured with an Epoch microplate spectrophotometer (BioTek, USA) by recording the absorbance at 550 nm. A 0.1% Triton X-100 solution was used as the positive control (100% of hemolysis) and pure PBS as the negative (0% of hemolysis). Minimum hemolytic concentration (MHC) was taken as the lowest concentration which induced 10% of hemolysis.

### Removal of trifluoroacetic acid counter ion and FTIR spectroscopy

Removal of trifluoroacetate counter ions was performed on VariPure IPE exchange columns. Before applying lipopeptides, ion exchange devices were conditioned as follows: 2 × 30 mL MeOH, 2 × 30 mL ACN, and 2 × 30 mL purified water. Previously lyophilized lipopeptides were dissolved in 10% acetonitrile and applied to ion exchange columns. Lipopeptides were eluted from the ion-exchange resin using a clean matrix. Thus obtained counter ion-free eluates were diluted with purified water and then subjected to a freeze-drying process. In order to confirm the absence of trifluoroacetate counter ions in the synthesized cationic lipopeptides, infrared spectroscopic studies were carried out. Infrared spectra were recorded on a Jasco FT/IR-410 spectrophotometer (Labor and Datentechnik GmbH, Germany) using the KBr tablet method. Tablets were prepared from 100 mg of potassium bromide (KBr) and 1.5 mg of the tested lipopeptide.

## Results

### Characterization of bacteriostatic properties

The antimicrobial tests indicated that lipopeptides containing hexadecanoic acid residue reveal high antibacterial activity toward Gram positives and Gram negatives; however, this activity is higher toward Gram positive bacteria (Tables 2, 3). The most potent bacteriostatic effect was observed with analogs containing more than one basic amino acid, that is C_16_-KK-NH_2_, C_16_-KKK-NH_2_, C_16_-KKKK-NH_2_, C_16_-KGK-NH_2_, and C_16_-KGKG-NH_2_. It was demonstrated that lipopeptides inhibit the growth of G+ and G− in the concentration range of 4–16 μg/mL. The exception is the *P. vulgaris* strain, which is resistant to the most active palmitate analogs and its MIC value reaches 512 μg/mL for all lipopeptide analogs. The lowest growth inhibition of G− was recorded for lipopeptides containing one basic amino acid: C_16_-K-NH_2_ and C_16_-KG-NH_2_. Results obtained for lipopeptides modified with tetradecanoic acid varied depending on the tested strain. The compounds exhibited relatively high antibacterial activity toward *B. subtilis* and *S. epidermidis*. The strongest bacteriostatic effect, from 4 to 8 μg/mL, was observed for the analogs C_14_-KK-NH_2_ and C_14_-KKK-NH_2_. Moderate bacteriostatic activity against the remaining Gram positive bacteria and Gram negatives was found (Tables 2, 3). However the activity toward gram-positive strains was higher in comparison to gram-negative strains. Lipopeptides modified with dodecanoic acid revealed rather poor bacteriostatic activity against tested G+ and G− strains (Tables 2, 3). Among all of the dodecanoic analogs, the highest growth inhibitory activity was perceived for lipopeptides containing one basic amino acid, i.e., C_12_-K-NH_2_ or C_12_-KG-NH_2_. MIC values determined for these compounds toward G+ and G− are in the range of 128–256 μg/mL, but for the strain *P. vulgaris* MIC was found at 512 μg/mL. Lipopeptides with two and three lysine residues (C_12_-KK-NH_2_, C_12_-KKK-NH_2_) are characterized by irregular activity. They are bacteriostatic to *S. epidermidis* and *B. subtillis* at a concentration of 64 μg/mL, but to *S. aureus* at a concentration of 2 and 1 mg/mL, respectively. The compounds are practically inactive against strains G−. Lipopeptides modified with decanoic acid are characterized by either poor bacteriostatic activity against G+ and G− strains or a complete lack thereof (Tables 2, 3). Among these lipopeptides, some higher bacteriostatic effect against G+ was observed for analogs C_10_-KKK-NH_2_ and C_10_-KKKK-NH_2_. They are also moderately active toward *S. epidermidis* and *B. subtillis*, while *S. aureus* and *E. fecalis* strains revealed complete resistance to them. None of the obtained lipopeptides modified with decanoic acid inhibited the growth of tested Gram negatives. Only the C_10_-K-NH_2_ analog exhibited very poor activity toward the *P. aeruginosa* strain; MIC value was observed at 512 μg/mL. Lipopeptides with attached octanoic fatty acid are generally inactive against gram positives as well as gram negatives. Among the 7 synthesized compounds, bacteriostatic activity at the concentration of 256 μg/mL toward *S. epidermidis* was noticed for C_8_-KKK-NH_2_ and C_8_-KGK-NH_2_. Additionally, C_8_-KGK-NH_2_ was bacteriostatic to *B. subtillis* at the concentration of 512 μg/mL.

**Table 2:** **Antimicrobial activity toward Gram positive strains**.

**Lipopeptides**	***Staphylococcus aureus***		***Staphylococcus epidermidis***		***Bacillus subtillis***		***Enterococcus fecalis***	
	**MIC**	**MBC**	**MIC**	**MBC**	**MIC**	**MBC**	**MIC**	**MBC**
C_16_-K-NH_2_	512	512	8	16	8	8	32	64
C_16_-KK-NH_2_	8	8	4	4	4	4	8	8
C_16_-KKK-NH_2_	8	8	4	4	8	8	16	16
C_16_-KKKK-NH_2_	4	8	4	4	4	4	16	16
C_16_-KG-NH_2_	8	8	4	4	4	4	16	16
C_16_-KGK-NH_2_	8	8	4	4	4	4	8	16
C_16_-KGKG-NH_2_	16	32	4	4	4	4	16	16
C_14_-K-NH_2_	64	64	16	16	32	32	64	64
C_14_-KK-NH_2_	64	64	4	4	4	4	32	64
C_14_-KKK-NH_2_	64	64	8	8	4	4	64	64
C_14_-KKKK-NH_2_	128	128	16	16	4	4	64	64
C_14_-KG-NH_2_	32	32	16	16	16	16	32	32
C_14_-KGK-NH_2_	64	128	16	16	8	8	64	64
C_14_-KGKG-NH_2_	64	128	32	32	16	16	64	64
C_12_-K-NH_2_	256	256	128	128	128	256	256	256
C_12_-KK-NH_2_	2·10^3^	2·10^3^	64	64	64	128	512	512
C_12_-KKK-NH_2_	1·10^3^	1·10^3^	64	64	64	128	512	512
C_12_-KKKK-NH_2_	1·10^3^	1·10^3^	64	64	128	256	512	512
C_12_-KG-NH_2_	256	512	128	128	128	256	256	256
C_12_-KGK-NH_2_	>2·10^3^	1·10^3^	64	128	512	1·10^3^	2·10^3^	>2·10^3^
C_12_-KGKG-NH_2_	2·10^3^	2·10^3^	256	512	64	64	512	512
C_10_-K-NH_2_	1·10^3^	>1·10^3^	1·10^3^	1·10^3^	1·10^3^	1·10^3^	>2·10^3^	>2·10^3^
C_10_-KK-NH_2_	>2·10^3^	>2·10^3^	256	512	256	512	>2·10^3^	>2·10^3^
C_10_-KKK-NH_2_	>2·10^3^	>2·10^3^	128	256	128	256	>2·10^3^	>2·10^3^
C_10_-KKKK-NH_2_	>2·10^3^	>2·10^3^	64	128	128	128	>2·10^3^	>2·10^3^
C_10_-KG-NH_2_	>2·10^3^	>2·10^3^	512	1·10^3^	512	512	1·10^3^	>1·10^3^
C_10_-KGK-NH_2_	>2·10^3^	>2·10^3^	512	512	512	1·10^3^	>2·10^3^	>2·10^3^
C_10_-KGKG-NH_2_	>2·10^3^	>2·10^3^	512	512	512	1·10^3^	2·10^3^	>2·10^3^
C_8_-K-NH_2_	>1·10^3^	>1·10^3^	>1·10^3^	>1·10^3^	>1·10^3^	>1·10^3^	>1·10^3^	>1·10^3^
C_8_-KK-NH_2_	>1·10^3^	>1·10^3^	>1·10^3^	>1·10^3^	>1·10^3^	>1·10^3^	>1·10^3^	>1·10^3^
C_8_-KKK-NH_2_	>2·10^3^	>2·10^3^	>256	512	1·10^3^	1·10^3^	>2·10^3^	>2·10^3^
C_8_-KKKK-NH_2_	>2·10^3^	>2·10^3^	>2·10^3^	>2·10^3^	>2·10^3^	>2·10^3^	2·10^3^	>2·10^3^
C_8_-KG-NH_2_	>2·10^3^	>2·10^3^	>2·10^3^	>2·10^3^	>2·10^3^	>2·10^3^	>2·10^3^	>2·10^3^
C_8_-KGK-NH_2_	>2·10^3^	>2·10^3^	256	1·10^3^	512	1·10^3^	>2·10^3^	>2·10^3^
C_8_-KGKG-NH_2_	>2·10^3^	>2·10^3^	>2·10^3^	>2·10^3^	>2·10^3^	>2·10^3^	>2·10^3^	>2·10^3^

*MIC and MBC are expressed as μg/mL*.

**Table 3:** **Antimicrobial activity toward Gram negative strains**.

**Lipopeptides**	***Escherichia coli***		***Klebsiella pneumoniae***		***Pseudomonas aeruginosa***		***Proteus vulgaris***	
	**MIC**	**MBC**	**MIC**	**MBC**	**MIC**	**MBC**	**MIC**	**MBC**
C_16_-K-NH_2_	512	>512	512	>512	512	>512	>512	>512
C_16_-KK-NH_2_	8	8	16	16	8	8	>512	>512
C_16_-KKK-NH_2_	8	16	4	4	8	8	>512	>512
C_16_-KKKK-NH_2_	8	8	4	8	8	8	>512	>512
C_16_-KG-NH_2_	256	256	128	256	256	1·10^3^	>512	>512
C_16_-KGK-NH_2_	8	8	16	16	16	32	>512	>512
C_16_-KGKG-NH_2_	16	16	16	16	8	8	>512	>512
C_14_-K-NH_2_	128	128	64	512	128	1·10^3^	>512	>512
C_14_-KK-NH_2_	64	128	128	128	32	64	>512	>512
C_14_-KKK-NH_2_	128	128	128	128	32	128	>512	>512
C_14_-KKKK-NH_2_	256	256	128	256	256	512	>512	>512
C_14_-KG-NH_2_	64	64	32	32	32	64	>512	>512
C_14_-KGK-NH_2_	128	128	64	64	32	64	>512	>512
C_14_-KGKG-NH_2_	128	128	128	128	64	128	>512	>512
C_12_-K-NH_2_	256	256	256	256	128	256	>512	>1·10^3^
C_12_-KK-NH_2_	2·10^3^	2·10^3^	2·10^3^	2·10^3^	2·10^3^	2·10^3^	>2·10^3^	>2·10^3^
C_12_-KKK-NH_2_	>2·10^3^	1·10^3^	1·10^3^	1·10^3^	512	1·10^3^	>2·10^3^	>2·10^3^
C_12_-KKKK-NH_2_	256	256	>2·10^3^	1·10^3^	2·10^3^	2·10^3^	>2·10^3^	>2·10^3^
C_12_-KG-NH_2_	256	512	256	512	256	512	>1·10^3^	>2·10^3^
C_12_-KGK-NH_2_	>2·10^3^	1·10^3^	>2·10^3^	1·10^3^	1·10^3^	1·10^3^	>2·10^3^	>2·10^3^
C_12_-KGKG-NH_2_	2·10^3^	2·10^3^	2·10^3^	2·10^3^	1·10^3^	1·10^3^	>2·10^3^	>2·10^3^
C_10_-K-NH_2_	1·10^3^	1·10^3^	1·10^3^	1·10^3^	512	1·10^3^	>2·10^3^	>2·10^3^
C_10_-KK-NH_2_	>2·10^3^	>2·10^3^	>2·10^3^	>2·10^3^	2·10^3^	2·10^3^	>2·10^3^	>2·10^3^
C_10_-KKK-NH_2_	>2·10^3^	>2·10^3^	>2·10^3^	>2·10^3^	>2·10^3^	>2·10^3^	>2·10^3^	>2·10^3^
C_10_-KKKK-NH_2_	>2·10^3^	>2·10^3^	>2·10^3^	>2·10^3^	>2·10^3^	>2·10^3^	>2·10^3^	>2·10^3^
C_10_-KG-NH_2_	>2·10^3^	>2·10^3^	1·10^3^	2·10^3^	1·10^3^	2·10^3^	>2·10^3^	>2·10^3^
C_10_-KGK-NH_2_	>2·10^3^	>2·10^3^	>2·10^3^	>2·10^3^	2·10^3^	2·10^3^	>2·10^3^	>2·10^3^
C_10_-KGKG-NH_2_	>2·10^3^	>2·10^3^	2·10^3^	2·10^3^	1·10^3^	2·10^3^	>2·10^3^	>2·10^3^
C_8_-K-NH_2_	>1·10^3^	>1·10^3^	>1·10^3^	>1·10^3^	>2·10^3^	>2·10^3^	>2·10^3^	>2·10^3^
C_8_-KK-NH_2_	>1·10^3^	>1·10^3^	>1·10^3^	>1·10^3^	>2·10^3^	>2·10^3^	>2·10^3^	>2·10^3^
C_8_-KKK-NH_2_	>2·10^3^	>2·10^3^	>2·10^3^	>2·10^3^	>2·10^3^	>2·10^3^	>2·10^3^	>2·10^3^
C_8_-KKKK-NH_2_	>1·10^3^	>1·10^3^	>2·10^3^	>2·10^3^	>2·10^3^	>2·10^3^	>2·10^3^	>2·10^3^
C_8_-KG-NH_2_	>2·10^3^	>2·10^3^	>2·10^3^	>2·10^3^	>2·10^3^	>2·10^3^	>2·10^3^	>2·10^3^
C_8_-KGK-NH_2_	>2·10^3^	>2·10^3^	>2·10^3^	>2·10^3^	>2·10^3^	>2·10^3^	>2·10^3^	>2·10^3^
C_8_-KGKG-NH_2_	>2·10^3^	>2·10^3^	>2·10^3^	>2·10^3^	>2·10^3^	>2·10^3^	>2·10^3^	>2·10^3^

*MIC and MBC are expressed as μg/mL*.

### Characterization of bactericidal properties

The highest bactericidal activity was revealed for lipopeptides modified with hexadecanoic acid. C_16_-KK-NH_2_, C_16_-KKK-NH_2_, C_16_-KKKK-NH_2_, C_16_-KGK-NH_2_, and C_16_-KGKG-NH_2_ are bactericidal to G+ and G− in a concentration range similar to MIC, i.e., from 4 to 32 μg/mL. The bactericidal activity of the analogs containing one lysine residue (C_16_-K-NH_2_ or C_16_-KG-NH_2_) is distinctly lower. Although, *S. epidermidis, B. subtillis* and *E. fecalis* are already sensitive to these at a concentration of 4–64 μg/mL; unlike *S. aureus*, which is sensitive only at 512 μg/mL. For G− bacteria, much weaker bactericidal activity was observed. MBC values of compounds where the total net charge of the molecule is +1 reach 256–1·10^3^ μg/mL. Selected myristates as well as palmitates exhibit good bactericidal activity. The obtained MBC values were usually as much as twice as high as the MICs. The strongest bactericidal effect was proven for analogs of C_14_-KK-NH_2_ and C_14_-KKK-NH_2_. The most sensitive strains are *B. subtillis* and *S. epidermidis*. MBC determined for these organisms is within the range of 4–8 μg/mL. MBC of the lipopeptides C_12_-K-NH_2_ and C_12_-KG-NH_2_ is only the same as MIC for *S. epidermidis* and *E. fecalis*. For strains of *S. aureus* and *B. subtillis*, MBC is twice as high as MIC. Lipopeptides containing two or three lysine residues (C_12_-KK-NH_2_, C_12_-KKK-NH_2_) are weakly bactericidal to all of the studied gram negatives. Most susceptible to C_12_-KKK-NH_2_ was *P. aeruginosa*; MBC was observed at 512 μg/mL. None of the decanoic analogs have any bactericidal activity against *S. aureus* and *E. fecalis*. Strains of *S. epidermidis* and *B. subtillis* are more sensitive, in particular to C_10_-KKK-NH_2_ and C_10_-KKKK-NH_2_, and MBC values determined for these compounds range 128–256 μg/mL. From MBC values it can be concluded that decanoic modified lipopeptides are not bactericidal toward the tested strains. The MBC test demonstrated that only C_8_-KKK-NH_2_ was bactericidal to *S. epidermidis* at the concentration of 512 μg/mL.

### Characterization of antifungal properties

It was found that palmitic acid derived peptides are weakly active against *Candida* species and *Aspergillus* molds (Table 4). This was confirmed by MIC and MFC determination. Of the seven analogs, the best fungistatic properties were confirmed for C_16_-KK-NH_2_, C_16_-KKK-NH_2_, and C_16_-KKKK-NH_2_. Both the MIC and the MFC are 128 μg/mL. Analogs containing glycine in the sequence (i.e., C_16_-KG-NH_2_, C_16_-KGK-NH_2_, and C_16_-KGKG-NH_2_) show weaker activity against *C. tropicalis* and *A. brasiliensis*. The lowest antifungal activity among all hexadecanoic analogs was revealed for lipopeptides containing one lysine: C_16_-K-NH_2_. Lipopeptides modified with tetradecanoic acid revealed some fungistatic and fungicidal activity against *Candida* and *Aspergillus* species. The lowest MIC and MBC value, i.e., 64 μg/mL, toward *A. brasiliensis* was noted in two cases: C_14_-KKK-NH_2_ and C_14_-KKKK-NH_2_. These compounds inhibit the growth of *C. albicans* and *C. tropicalis* slightly less effectively, because they require a concentration twice as high—128 μg/mL (Table 4). It was found that, at the same concentrations, all the tetradecanoic lipopeptides are fungistatic and fungicidal to the tested strains of *C. albicans, C. tropicalis* and *A. brasiliensis*. It was observed that dodecanoic lipopeptides weakly inhibits the growth of yeasts of *Candida* and *A. brasiliensis* (Table 4). The most bacteriostatically efficient compound proved to be that containing four lysines—C_12_-KKKK-NH_2_; MIC of this compound is in the range 128–256 μg/mL. Nearly identical properties were demonstrated by C_12_-K-NH_2_. Other analogs did not show significant bacteriostatic activity toward the tested fungi species. The best MFC results were observed for C_12_-KKKK-NH_2_. Analogs C_12_-K-NH_2_ and C_12_-KG-NH_2_ presented the same fungicidal activity—512 μg/mL. Generally, decanoic lipopeptides revealed no fungistatic activity toward *Candida* species and *A. brasiliensis* (Table 4). Some very weak fungistatic effect at the concentration of 512 μg/mL was noticed for C_10_-KGK-NH_2_ and C_10_-KGKG-NH_2_ toward *C. tropicalis* and for C_10_-K-NH_2_ toward *A. brasiliensis*. All tested fungi were totally resistant to octanoic analogs, as can be seen from the high MIC and MFC values. Results of antimicrobial studies are presented in Tables 2–4.

**Table 4:** **Antimicrobial activity toward fungi strains and toxicity toward human red blood cells**.

**Lipopeptides**	***Candida albicans***		***Candida tropicalis***		***Aspergillus brasiliensis***		**Hemolysis**
	**MIC**	**MFC**	**MIC**	**MFC**	**MIC**	**MFC**	**HC_10_**
C_16_-K-NH_2_	512	512	>512	>512	>512	>512	512
C_16_-KK-NH_2_	128	128	128	128	128	128	16
C_16_-KKK-NH_2_	128	128	128	128	128	128	32
C_16_-KKKK-NH_2_	128	128	128	128	128	128	16
C_16_-KG-NH_2_	128	128	256	512	512	512	256
C_16_-KGK-NH_2_	128	128	256	256	128	128	64
C_16_-KGKG-NH_2_	128	128	256	256	128	128	64
C_14_-K-NH_2_	512	512	>512	>512	512	512	512
C_14_-KK-NH_2_	128	128	256	256	256	256	128
C_14_-KKK-NH_2_	128	128	128	128	64	64	256
C_14_-KKKK-NH_2_	128	128	128	128	64	64	256
C_14_-KG-NH_2_	128	128	256	256	128	128	>1·10^3^
C_14_-KGK-NH_2_	128	128	256	256	128	128	128
C_14_-KGKG-NH_2_	128	128	512	512	128	128	512
C_12_-K-NH_2_	256	512	256	512	256	512	1·10^3^
C_12_-KK-NH_2_	1·10^3^	2·10^3^	1·10^3^	2·10^3^	2·10^3^	2·10^3^	>4·10^3^
C_12_-KKK-NH_2_	1·10^3^	1·10^3^	1·10^3^	1·10^3^	512	512	512
C_12_-KKKK-NH_2_	256	256	256	512	128	256	1·10^3^
C_12_-KG-NH_2_	512	512	512	512	256	512	1·10^3^
C_12_-KGK-NH_2_	2·10^3^	2·10^3^	2·10^3^	2·10^3^	1·10^3^	2·10^3^	1·10^3^
C_12_-KGKG-NH_2_	1·10^3^	2·10^3^	1·10^3^	2·10^3^	1·10^3^	1·10^3^	256
C_10_-K-NH_2_	1·10^3^	2·10^3^	1·10^3^	2·10^3^	512	1·10^3^	>4·10^3^
C_10_-KK-NH_2_	2·10^3^	2·10^3^	1·10^3^	1·10^3^	1·10^3^	1·10^3^	4·10^3^
C_10_-KKK-NH_2_	2·10^3^	2·10^3^	2·10^3^	2·10^3^	1·10^3^	2·10^3^	>4·10^3^
C_10_-KKKK-NH_2_	>2·10^3^	>2·10^3^	2·10^3^	2·10^3^	2·10^3^	2·10^3^	>4·10^3^
C_10_-KG-NH_2_	1·10^3^	2·10^3^	1·10^3^	2·10^3^	1·10^3^	2·10^3^	>4·10^3^
C_10_-KGK-NH_2_	1·10^3^	1·10^3^	512	1·10^3^	1·10^3^	1·10^3^	>4·10^3^
C_10_-KGKG-NH_2_	1·10^3^	2·10^3^	512	2·10^3^	1·10^3^	2·10^3^	>4·10^3^
C_8_-K-NH_2_	1·10^3^	1·10^3^	1·10^3^	2·10^3^	1·10^3^	1·10^3^	>4·10^3^
C_8_-KK-NH_2_	1·10^3^	1·10^3^	1·10^3^	1·10^3^	1·10^3^	1·10^3^	>4·10^3^
C_8_-KKK-NH_2_	2·10^3^	2·10^3^	2·10^3^	2·10^3^	2·10^3^	2·10^3^	>4·10^3^
C_8_-KKKK-NH_2_	2·10^3^	2·10^3^	2·10^3^	2·10^3^	2·10^3^	2·10^3^	>4·10^3^
C_8_-KG-NH_2_	2·10^3^	2·10^3^	2·10^3^	2·10^3^	2·10^3^	2·10^3^	>4·10^3^
C_8_-KGK-NH_2_	2·10^3^	2·10^3^	2·10^3^	2·10^3^	2·10^3^	2·10^3^	>4·10^3^
C_8_-KGKG-NH_2_	2·10^3^	2·10^3^	2·10^3^	2·10^3^	2·10^3^	2·10^3^	>4·10^3^

*MIC, MFC and MHC are expressed as μg/mL*.

*HC_10_ – concentration of lipopeptide inducing 10% of hemolysis*.

### Influence of the presence of TFA on the antimicrobial activity

The most promising compounds, i.e., lipopeptides containing palmitic and myristic fatty acid residues, were tested after removal of counter ions to determine whether this influences their antimicrobial activity. We noticed some differences in the activity of compounds toward various strains. The determined antimicrobial activity was usually either the same for compounds with and without TFA or 2-fold higher/ lower, depending on the compounds and strain tested. For *S. aureus*, palmitic analogs without TFA (with the exception of C_16_-K-NH_2_) were twice as active as compounds containing TFA in both MIC and MBC assays. MICs obtained for C_14_-KK-NH_2_ and C_14_-KKK-NH_2_ were also twice as low as those obtained for lipopeptides after TFA removal. For the compounds C_14_-KGK-NH_2_ and C_14_-KGKG-NH_2_ the same difference was observed, but for the MBC values. Similar results were obtained with the *E. faecalis* strain. For almost all palmitic analogs without counter ions, the bacteriostatic and bactericidal concentrations were twice as low as peptides containing TFA. For myristic analogs, we did not observe any discrepancies in the activity of compounds both before and after removal of the counter ion. Bactericidal activity of the majority of palmitic analogs without TFA against *E. coli* was twice as high as peptides with a counter ion. MIC values were different for palmitic analogs containing glycine and more than one lysine residues. Bacteriostatic activity was also higher for myrisitic analogs: C_14_-K-NH_2_, C_14_-KK-NH_2_, and C_14_-KKK-NH_2_. For *K. pneumoniae*, differences between the activity of the compounds after counter ion removal were noticed only for a few compounds. Obtained MBCs were lower for lipopeptides without TFA, i.e., C_16_-K-NH_2_, C_16_-KKKK-NH_2_, and C_14_-KKKK-NH_2_, while for compounds C_16_-KGK-NH_2_, C_16_-KGKG-NH_2_, and C_14_-KG-NH_2_ we observed a decrease in antibacterial activity after removal of TFA. A decrease in the activity of compounds without counter ions was observed for the majority of the tested myristic analogs in MIC and MBC tests with *C. albicans*.

### Hemolysis

Lytic activity against human erythrocytes was measured to assess the toxicity of lipopeptides to eukaryotic cells. Lipopeptides containing a hexadecanoic chain displayed strong hemolysis. The strongest toxicity was demonstrated by palmitic derivatives containing more than one lysine residue: C_16_-KK-NH_2_, C_16_-KKKNH_2_, and C_16_-KKKK-NH_2_ (MHC 16–32 μg/mL). Compounds with the same cationic charge additionally containing a glycine residue showed slightly reduced lytic activity (MHC 64 μg/mL). Among the group of lipopeptides with a tetradecanoic acid, two analogs—C_14_-KK-NH_2_ and C_14_-KGK-NH_2_—were established as moderately hemolytic. These lead to lysis of red blood cells at the concentration of 128 μg/mL. Compounds containing 3 or 4 basic amino acid destroy erythrocytes at a higher concentration, i.e., only at 256 μg/mL. The rest of the synthesized lipopeptides are very weakly hemolytic or display no hemolysis at all within the range of the tested concentrations. The study shows that the hemolytic activity of lipopeptides increases with the length of the attached hydrophobic chain. Among the synthesized lipopeptides, the greatest hemolytic activity was observed for hexadecanoic modified lipopeptides. For those lipopeptides comprising a fragment of octanoic acid, hemolysis was not observed, even at a concentration of 4·10^3^ μg/mL. Furthermore, within the group of lipopeptides with a hexadecanoic fatty acid a strong dependence of the hemolytic activity on amino acid composition of the hydrophilic portion of the molecule was observed. Compounds endowed with a greater positive charge were more toxic to human erythrocytes.

### TFA removal and antibacterial activity of lipopeptides without a counter ion

**Figure 1:**
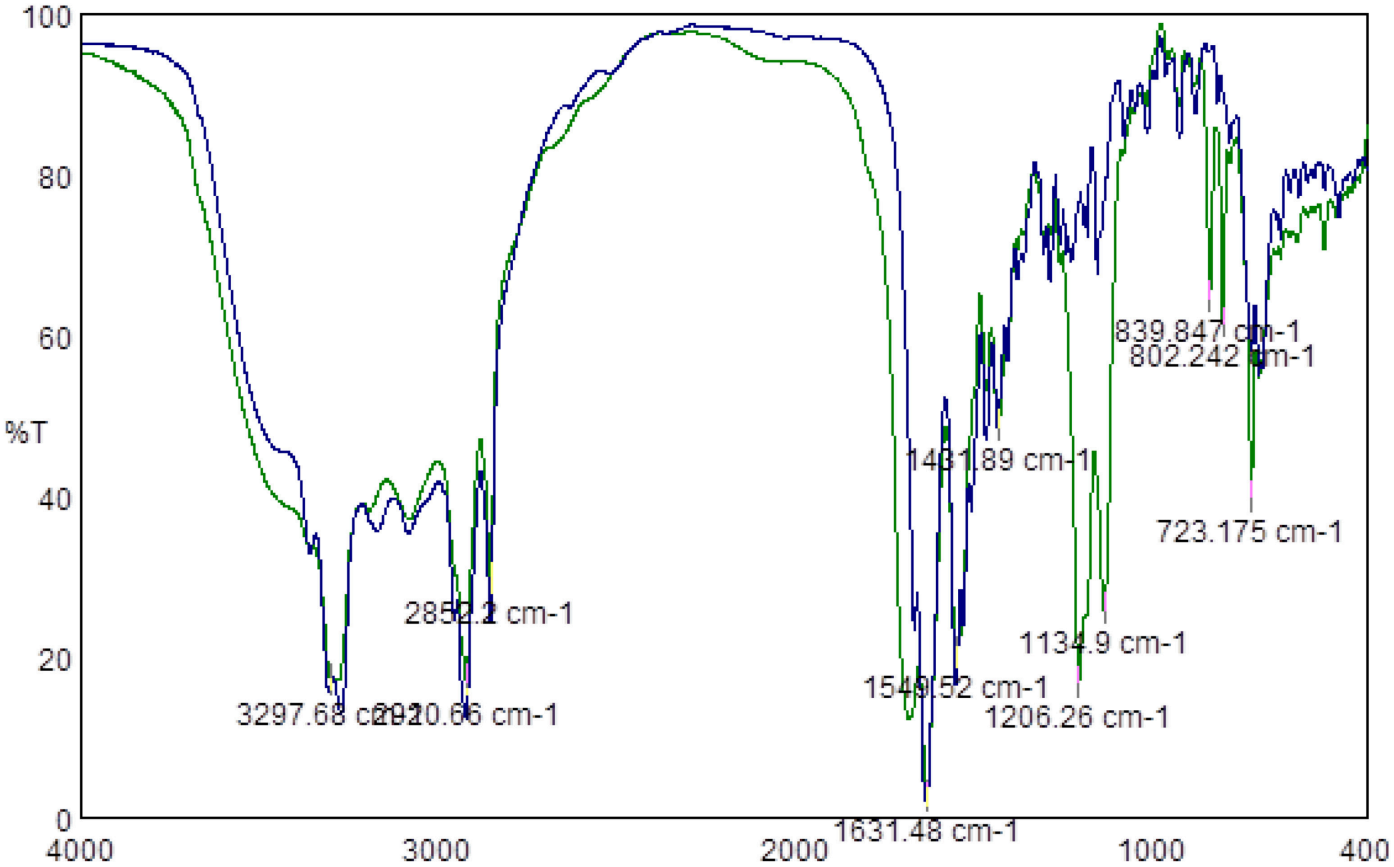
**Example of FTIR spectra of lipopeptide C_16_-KK-NH_2_ before TFA counter ion removal (Green Line) and after TFA counter ion removal (blue line)**.

Since the most significant antimicrobial activities were observed for lipopeptides containing hexadecanoic and tetradecanoic hydrocarbon chains, only those two groups of compounds were subjected to TFA counter ion removal to test the influence of TFA presence on the antimicrobial activity of lipopeptides themselves. Obtained FTIR spectra confirmed the absence of TFA counter ions, thus proving that the process of removing the counter ions via a VariPure IPE ion-exchange column ran properly. Figure 1 presents IR spectra of lipopeptides before (green line) and after (blue line) TFA removal. There are visible bands derived from the stretching vibration of CF_2_ and CF_3_ groups within the range of 1350–1120 cm^−1^ (green line). However, there is no such band in the sample subjected to removal of the counter ion.

## Discussion

The antimicrobial activity of synthetic cationic lipopeptides is widely studied and many structures have already been described. For example, ultrashort lipopeptides very similar to ours, composed of lysine, glycine, alanine, leucine and isoleucine acylated with dodecanoic, tetradecanoic, and hexadecanoic acids were synthesized. For each of the ultrashort lipopeptides, one amino acid of D-configuration was introduced. The best MIC values were obtained for the compound C_16_-KGG-[D]-K-NH_2_. This was found to be active toward G− and G+ tested strains (Makovitzki et al., 2006). We obtained similar results for palmitic derivatives. The compounds exhibited very potent antibacterial activity, but unfortunately they turned out to be also the most toxic to human erythrocytes. Due to their hemolytic activity only topical application can be considered. However, in some previous studies the compound C_16_-KK-NH_2_ exhibited toxicity toward human keratinocytes HaCaT exposed to its microbiologically active concentrations (Barańska-Rybak et al., 2013). Therefore, further consideration of this compound should be as a topical agent applied on uninjured skin, e.g., eradication of bacterial colonization, or as a preservative for dermatological drugs or cosmetics. For abovementioned ultrashort lipopeptides, similarly to our results, strong hemolytic activity, at the level of 8% at 20 μM, was observed. It seems that these lipopeptides do not discriminate effectively between bacterial and eukaryotic cells. Some better results have been obtained for myristic residues. We observed some discrimination between various microbial species, which would suggest some additional mechanism of antibacterial action. However, the toxicity is still at a relatively high level and to improve the selectivity of the lipopeptide antibiotic structure modifications are needed. A possible solution is also the use of a combination of active lipopeptides with conventional antibiotics, which would probably allow the use of lower concentrations of both substances (Barchiesi et al., 2007; Cirioni et al., 2007; Simonetti et al., 2009). According to the literature, the anti-infective activity of C_16_-KK-NH_2_ has been studied utilizing the rat model, where the compound demonstrated effectiveness against induced staphylococcal infections, at the same time without causing harmful side effects in the test animals. Kamysz et al. studied the activity of C_16_-KK-NH_2_ and C_16_-KK-OH against gram-positive cocci resistant to methicillin and vancomycin. The tested compounds showed high antimicrobial activity against these strains and a slightly synergistic effect in combination with amoxicillin, imipenem and vancomycin (Kamysz et al., 2007). The antibacterial activity of selected lipopeptides was also determined against clinical isolates of *S. aureus*. The tested compounds, i.e., C_16_-KK-NH_2_, C_16_-KKK-NH_2_, C_16_-KKKK-NH_2_, C_16_-KG-NH_2_ and C_16_-KGK-NH_2_, showed high antistaphylococcal activity against all tested strains, including those resistant to chloramphenicol, erythromycin and penicillin. G. Sarig et al. received a lipopeptide composed of three residues of lysine and two hydrocarbon chains (C_12_-KK-C_12_-K-NH_2_), wherein one of the hydrophobic chains separated the amino acid sequence into two parts. For this compound, a very high antimicrobial activity against *S. aureus* strains was found (2.5–5 μg/mL) and a weaker one against *E. coli* (≥ 40 μg/mL) (Sarig et al., 2010).

There is not much available data in the literature regarding the impact of counter ions on the biological activity of antimicrobial peptides obtained *via* chemical synthesis. However, Gaussier et. al. revealed that replacement of trifluoroacetic acid with HCl counter ions did not significantly affect the antimicrobial activity of pediocin PA-1 (Gaussier et al., 2002). In our study, the removal of TFA either caused no change in activity or the results differed by one well (Table 5). In the majority of cases, the obtained MICs and MBCs were lower for lipopeptides without counter ions. This might be a result of the different molecular weights of the two peptide forms, rather than being a direct influence on antimicrobial action. However, in a few cases peptides containing TFA were slightly more active toward *K. pneumoniae* and *C. albicans*, which would suggest some positive influence on antimicrobial activity. However, further study with the use of other counter ions and more microbial species is needed to clarify this issue.

**Table 5:** **Antimicrobial activity of lipopeptides without TFA counter ion, expressed as minimum inhibitory concentration and minimum bactericidal/fungicidal concentration**.

**Lipopeptides**	***Staphylococcus aureus***		***Enterococcus fecalis***		***Escherichia coli***		***Klebsiella pneumoniae***		***Candida albicans***	
	**MIC**	**MBC**	**MIC**	**MBC**	**MIC**	**MBC**	**MIC**	**MBC**	**MIC**	**MFC**
C_16_-K-NH_2_	128	128	32	32	512	512	512	512	512	512
C_16_-KK-NH_2_	4	4	4	4	4	4	16	32	256	256
C_16_-KKK-NH_2_	4	8	8	8	4	8	4	4	128	128
C_16_-KKKK-NH_2_	4	4	4	8	8	8	4	4	128	128
C_16_-KG-NH_2_	4	4	4	8	256	256	128	256	128	128
C_16_-KGK-NH_2_	4	4	4	4	4	4	32	64	128	128
C_16_-KGKG-NH_2_	8	16	16	16	8	8	16	32	128	128
C_14_-K-NH_2_	64	64	64	64	64	128	64	512	512	512
C_14_-KK-NH_2_	32	64	32	32	32	64	128	128	256	256
C_14_-KKK-NH_2_	32	32	32	64	128	128	128	256	256	256
C_14_-KKKK-NH_2_	128	128	64	64	256	256	128	128	256	256
C_14_-KG-NH_2_	32	32	32	32	64	64	32	64	256	256
C_14_-KGK-NH_2_	64	64	64	64	128	128	64	64	128	128
C_14_-KGKG-NH_2_	64	64	64	64	128	128	128	128	256	256

*MIC, MBC and MFC are expressed as μg/mL*.

## Conclusions

We have demonstrated that the antimicrobial activity of short cationic lipopeptides is strongly dependent on the net charge of the lipopeptide molecule. A higher antibacterial activity is displayed by lipopeptides with a net charge from +2 to +4 and a hexadecanoic fatty acid chain attached. This clearly suggests that a antimicrobial peptide molecule needs proper balance between a hydrophilic and hydrophobic entity to effectively interact with a bacterial membrane. Our studies also clarify that the presence of a TFA counter ion has no significant impact on the antimicrobial activity of the tested lipopeptides.

## Author contributions

KG: Conception and design of work, performing the experiments, collecting the literature, scientific description of the comments, corresponding author; MD: Co-operation in performing the experiments, collecting the literature, co-operation in the manuscript preparation; WK and WS: Co-operation in the manuscript preparation. All authors critically revised the manuscript and approved the final version.

### Conflict of interest statement

The authors declare that the research was conducted in the absence of any commercial or financial relationships that could be construed as a potential conflict of interest.

## Abbreviations

AMP: antimicrobial peptide
Boc: *tert*-butoxycarbonyl
CFU: colony forming units
CLSI: Clinical and Laboratory Standards Institute
DCM: dichloromethane
DIC: diisopropylcarbodiimide
DMF: *N,N*-dimethylformamide
Fmoc: 9-fluorenylmethoxycarbonyl
G−: Gram-negative bacteria
G+: Gram positive bacteria
HOBt: 1-hydroxybenzotriazole
MBC: minimum bactericidal concentration
MFC: minimum fungicidal concentration
MIC: minimum inhibitory concentration
TFA: trifluoroacetic acid
TIS: triisopropylosilane.
